# TLR9 expression in glioma tissues correlated to glioma progression and the prognosis of GBM patients

**DOI:** 10.1186/1471-2407-10-415

**Published:** 2010-08-10

**Authors:** Chao Wang, Shouqiang Cao, Ying Yan, Qiao Ying, Tao Jiang, Ke Xu, Anhua Wu

**Affiliations:** 1 Department of Neurosurgery, The First Affiliated Hospital of China Medical University, Shenyang, China; 2 Department of Biological Chemistry, China Medical University, Shenyang, China; 3 Central Lab, The First Affiliated Hospital of China Medical University, Shenyang, China; 4 Department of Neurosurgery, Beijing Tiantan Neurosurgical Institute, Beijing, China; 5 Department of Radiology, The First Affiliated Hospital of China Medical University, Shenyang, China

## Abstract

**Background:**

Our study aims to evaluate the expression of TLR9 in glioma tissues, examine the association between TLR9 expression, clinicopathological variables, and glioma patient outcome, we further characterized the direct effects of TLR9 agonist CpG ODN upon the proliferation and invasion of glioma cells *in vitro*.

**Methods:**

RT-PCR and immunofluorescence were used to determine the expression of TLR9 in glioma cell lines and clinical glioma samples. Tissue microarry and immunohistochemistry were applied to evaluated TLR9 expression in 292 newly diagnosed glioma and 13 non-neoplastic brain tissues. We further investigated the effect of CpG ODN on the proliferation and invasion of glioma cells *in vitro *with MTT assays and matrigel transwell assay respectively.

**Results:**

RT-PCR showed that TLR9 expressed in all the glioma samples and glioma cell lines we examined. The tissue array analysis indicated that TLR9 expression is correlated with malignancy of glioma (p < 0.01). Multivariate Cox regression analysis revealed that TLR9 expression is an independent prognostic factor for PFS of GBM patients(P = 0.026). TLR9 agonist CpG ODN has no significant effect on glioma proliferation, but matrigel transwell analysis showed that TLR9 agonist CpG ODN can significantly enhance glioma invasion *in vitro*.

**Conclusions:**

Our data indicated that TLR9 expression increases according to the histopathological grade of glioma, and the TLR9 expression level is related to the PFS of GBM patients. In addition, our findings warrant caution in the directly injection of TLR9 agonist CpG ODN  into glioma tissues for the glioma immunotherapy.

## Background

Glioma is the most common brain tumor, although with the combined treatment strategy including surgery, radiotherapy and chemotherapy, prognosis for high grade glioma especially glioblastoma multiform (GBM) altered little over the past 10 years, with a median survival only about 1 year[1], thus new treatment strategy are urgently needed. Immunotherapy is a promising treatment strategy for gliomas since the activated lymphocyte can specifically target and destroy tumor cells[2].

Recently, CpG based immunotherapy are intensively investigated. CpG is synthetic ODN with unmethylated CG dinucleotides within particular sequence contexts[3], CpG ODN has been used in clinical trials as a vaccine adjuvant for immunotherapy of cancer since it can mimic microbial DNA and activate immune system through the binding of TLR9. TLR9 belong to TLRs families which are evolutionarily well conserved transmembrane proteins that recognize microbe derived molecular patterns [4]. TLR9 resides in the endoplasmic reticulum and its activation results in increased production of inflammatory mediators[5,6], Except plasmacytoid DC and B cells, TLR9 can also expressed in breast[7], gastric[8], lung[9] and prostate[10] cancer cells.

Accumulating data indicated that TLR9 agonist CpG ODN can promote tumor development and metastasis[7,10-12]. We proposed that TLR9 signal pathway may also be important in glioma growth and progression. However its precise role in glioma remains unclear with studies supporting both glioma promotion and glioma inhibition results, there is report that local CpG immunotherapy can prolong the survival of mouse with glioma[13,14]. In contrast, Ginzkey et al[15] found increase in tumor size following intratumoral injection of immunostimulatory CpG ODN in a rat glioma model, this was consistent with our previous finding that the intratumoral injection of CpG ODN do not increase the survival time in GL261 glioma animal model[16], and these results indicated that directly local injection of CpG may not yield beneficial effects in glioma patients.

TLR9 expression has been examined in GBMs but not found to be of prognostic significance [17], however that investigation is at mRNA level, a protein level study is thus needed. The aim of this study was to evaluate the expression of TLR9 in a large series of glioma samples with tissue array and to examine the association between TLR9 expression, clinicopathological variables, and patient outcome. To further characterize the possible role of TLR9 expression on tumor cells, we examine the direct effects of CpG ODN upon the proliferation and invasion of glioma cells *in vitro*.

## Methods

### Cells and Cell culture

The human glioma cell lines U87-MG, U251-MG and rat glioma C6 cell lines were first obtained from ATCC and maintained in the Central Lab of First Hospital of China Medical University. Glioma cells were grown in Dulbecco's modified essential medium (DMEM, Gibcol, Gaithersburg, MD, USA) supplemented with 10% FBS (Hyclone Laboratories, Logan, USA), 100 U/ml penicillin, and 100 μg/ml streptomycin at 37°C with 5% CO_2 _in humidified air.

### CpG ODN and Cell challenge

Nuclease-resistant phosphorothioate-modified oligonucleotides (ODNs) were custom synthesized and purified by Takara Biotechnology (Dalian, China), The following sequences were used (the bold letters indicate the CpG motifs or the GpC motifs of the ODN): ODN 2006, T**CG **T**CG **TTT TGT **CG**T TTT GT**C G**TT; ODN 2006 Control, T**GC **T**GC **TTT TGT **GC**T TTT GT**G C**TT. The ODNs were dissolved in endotoxin free sterile water, aliquot and stored at -80°C until use.

### Immunofluorescence

U87-MG and U251-MG cells were grown on coverslips, after washing with serum free-DMEM, the cells were fixed for 10 min with 4% paraformaldehyde in PBS, permeabilized with 0.5% Triton X-100 in PBS and blocked 1 h in PBS supplemented with 5% FBS. Cells were incubated overnight at 4°C with TLR9 mAb (Img-305A, clone 26C593.2, Imgenex, San Diego, USA, 1:400 dilution), followed by Cy3-labelled anti-mouse secondary antibody (Sigma, USA, 1:400 dilution) for 2 h. The nuclei were counterstained with Hoechst (1:1000 dilutions). In the control samples, glioma cells were stained with secondary antibody and Hoechst, but without anti-TLR9 primary antibody. Results were observed and photographed under Leica confocal microscope.

### Tissue Samples and Patients

Tumor specimens were obtained from patients admitted for diagnosis and treatment to First Hospital of China Medical University and Beijing Tiantan Hospital. The diagnosis was made according to WHO criteria. The study was approved by the Ethics Committee of each institution and was based on the criteria of the Helsinki convention. Informed consent was obtained from each patient. Fresh surgical samples from glioma patients and non-neoplastic brain tissues(temporal lobectomy from epilepsy surgery) were immediately snap-frozen in liquid nitrogen upon surgical removal. One part of each sample was fixed with formalin, embedded with paraffin wax and kept at RT. The including criteria for the patients in this study are: 1) Patients do not have other cancers or diseases such as acute infection, diabetes and did not receive previous radiotherapy, chemotherapy, or corticosteroid therapy; 2). Patients had taken routine contrast-enhanced MRI examination before and after surgical operation; 3) Patients gave informed consent; 4). Patients who died from non-glioma-related cause were excluded. The extent of tumor resection was defined as follows: (0) residual tumor >30% or biopsy; (1) partial removal with residual tumor ≤ 30%; (2) gross total resection. Clinicopathological variables were retrospectively collected from medical records until July 2009. Progress-free survival (PFS) was defined from the date of surgery to the first MRI-confirmed recurrence or death, whichever occurred first.

For the survival analysis, to avoid the bias caused by different treatment strategy, in addition to the above screening criteria, patients must have received similar treatments: surgical resection performed by neurosurgeons who used similar operational techniques and principles. Every patient in the study received the same radiotherapy regimen. All Patients received chemotherapy after radiotherapy. The criteria ensured that the patient group was uniform, which strengthens the analysis. We stopped to collect data at July 2009, the observation for each patient will end once the patient is dead or have recurrent. 69 GBM patients fulfill these criteria and were used for the survival analysis. There are no patients survived more than 24 months.

### RNA Extraction and RT-PCR assay for detection of Toll-Like Receptor 9

Total RNA was extracted from 3 glioma cell lines and 34 clinical glioma samples using Trizol reagent (Invitrogen, USA), 1 ug RNA was used as template for cDNA Synthesis, cDNA was synthesized with First-Strand Synthesis System for RT-PCR Kit (Catalog No. 11904-018 Invitrogen). RT-PCR analyses were performed using the following primer sets, human TLR9(sense: 5'-TGTAATAACAGTTGCCGTCCAT-3', antisense: 5'-CAGCCTTTCCTTGTCCT CC-3', product size:344 bp), rat TLR9(sense: 5'-GGACAGTTCTCTCCACTCGC-3', antisense: 5'-TTCTTTGAGACGGGAGTGCT-3', product size: 509 bp), GAPDH(sense: 5'-TCCACCAC CCTGTTGCTGTA-3', antisense: 5'-ACCACAGTCCATGCCATCAC-3', product size:450 bp). GAPDH was used as housekeeping gene control, GADPH consensus primers were shared for *Homo sapiens *and *Rattus norvegicus*. Reactions were carried out in a Gradient Thermal Cycler (Biometra, Goettingen, Germany) for 30 cycles which consisted of 94.0°C 30 s, 55°C 30 s, 72°C 30 s. The reaction took place in a total volume of 25 ul. Products were analyzed by 1.5% agarose gel electrophoresis and visualized by Gene finder staining under ultraviolet. All primers were synthezed by Takara Biotechnology (Dalian, China). The expression of TLR9 in U87 and U251 cells were further confirmed by immunoflurescence analysis, and the expression of TLR9 in glioma tissues were further confirmed by immunohistochemistry method.

### Selection of Diffuse Glioma Samples and Construction of Glioma Tissue Microarray

The formalin-fixed, paraffin embedded archival tissue blocks were retrieved, and the matching HE-stained slides were screened for representative tumor regions by a neuropathologist. Glioma samples were then grouped according to the diagnostic criteria of the WHO 2000 classification system. The composition of tumors included in the tissue microarray is listed in Table 1. tissue microarray was constructed with tissue microarrayer (Beecher Instruments, Silver Springs, MD, USA) as described previously[18]. The tissue microarray included 292 primary gliomas representing different histological types and grades of glioma. Each tumor was sampled in duplicate from representative areas using a 0.6-mm punch, yielding composite array blocks comprising a total of 584 tissue cores. In addition, thirteen non-neoplastic brain tissues from epilepsy surgical resections were also included. Normal kidney samples provided by department of surgery were used as positive control.

**Table 1:** Composition of tumor types in glioma tissue array

gliomas	Number of cases (tissue cores)
Glioblastoma (GBM, WHO Grade IV)	128 (256)
Anaplastic astrocytoma (AA, WHO Grade III)	27 (54)
Diffuse astrocytoma (LGA, WHO Grade II)	33 (66)
Anaplastic oligodendroglioma (AO, WHO Grade III)	16 (32)
Oligodendroglioma (ODG, WHO Grade II)	26 (52)
Anaplastic mixed oligoastrocytoma (AMOA, WHO Grade III)	30 (60)
Oligoastrocytoma (MOA, WHO Grade II)	32 (64)
non neoplastic brain tissue(NB)	13 (26)

### Immunochemistry

Paraffin-embedded specimens were cutted into 4 um sections and baked at 65°C for 30 min. The sections were deparaffinized with xylenes and rehydrated. Sections were submerged into EDTA (PH = 8.0) and autoclaved for antigen retrieval, then treated with 3% hydrogen peroxide, followed by incubation with 1% FBS. Anti-TLR9 (Img-305A, clone 26C593.2, Imgenex, San Diego, USA, 1:100 dilutions) was added and incubated overnight at 4°C. For negative controls, the primary antibody was replaced by normal mouse serum. Human normal kidney samples were used as a positive control. Horseradish peroxidase (HRP) labeled secondary antibody in the MaxVision™ HRP-Polymer antimouse/rabbit IHC kit (KIT-5930 Maixin Biol, Fu Zhou, China) was applied and incubated for 30 mins at room temperature, followed by 5 mins incubation at room temperature with DAB provided in the kit for color development. The sections were finally counterstained with haematoxylin and mounted with Permount (BIOS, Beijing, China). Results were visualized and photographed under a light microscope (Olympus BX-51; Olympus Optical).

The degree of immunostaining of sections was viewed and scored separately by two independent investigators, the scores were determined by combining the proportion of positively stained tumor cells and the intensity of staining. Scores from the two investigators were averaged for further comparative evaluation of the TLR9 expression. The proportion of positively stained tumor cells was graded as follows: 0 (no positive tumor cells), 1(<10% positive tumor cells), 2 (10-50% positive tumor cells) and 3 (>50% positive tumor cells). The intensity of staining were recorded on a scale of 0 (no staining), 1 (weak staining, light yellow), 2(moderate staining, yellowish brown) and 3 (strong staining, brown). The staining index was calculated as follows: staining index = staining intensity × proportion of positively stained tumor cells. The staining index score > 4 was defined as tumors with high expression, and staining index ≤ 4 as tumors with low expression of TLR9.

### MTT assay

MTT assay was used to assess the effect of CpG ODN on glioma cells proliferation. Briefly, 1 × 10^4 ^U87, U251 and C6 cells were seeded into 96-well plates in triplicate in a volume of 100 ul per well, then incubated at 37°C for 24 h for cells adherence. CpG ODN(ODN 2006), ODN 2006 control(10 uM) and same volume of medium (10 uM) were added, cell growth was measured at 24, 48, 72 hours after the treatment. MTT (Sigma) was dissolved in PBS at 5 mg/ml and filtered to be sterilized, 20 ul MTT solution was added at different time points. Plates were then incubated at 37°C for 4 h, 100 ul dimethylsulfoxide was added to each well and mixed thoroughly to dissolve the blue-violet crystals. Cell viability data were measured with an ELISA reader (TECAN SUNRISE, Austria) at 490 nm.

### Transwell assay

The invasion assay was done in 24 well cell culture chambers using transwell inserts (Corning life sciences, NY, Bioscience, San Jose, CA, USA) with 8 um Pore membrane precotated with matrigel (BD biosciense, San Jose, CA, USA). U87 cells were plated at the density of 1 × 10^4 ^per upper well in 200 ul culture medium (DMEM, 1%FBS), the lower chamber was filled with 500 μl medium (DMEM, 20% FBS). ODN 2006 (10 uM), ODN 2006 control (10 uM), same volume of the control medium, or Chloroquine (10 uM) was added to both upper and lower well. The cells were allowed to invade for 24 hours, after which, the noninvading cells with Matrigel matrix were removed from the upper surface of the membrane by scrubbing with a cotton-tipped swab. Cells on the lower surface of the filter were fixed for 30 min in methanol and glacial acetic acid mixture (3:1), air-dried briefly, and stained with Giemsa. The number of invaded cells was counted from five preselected microscopic fields at 200× magnification, all experiments were performed in triplicate.

### Statistical analysis

SPSS statistical software for Windows 13.0 (SPSS, Inc., Chicago, Illinois, USA) was used for all statistical analysis. Differences of tumor cell proliferation and invasion between groups were analyzed by t-test. The Jonckheere-Terpstra test was used to correlate cumulative TLR9 expression with glioma grading, and the chi-square test was used for comparisons between groups. Cox regression was used to correlate TLR9 expression with PFS while adjusting for clinicopathological variables. Survival curves were constructed using the Kaplan-Meier method. The log-rank test was used for comparison between groups. P ≤ 0.05 was defined as significance.

## Results

### TLR9 is expressed in glioma cell lines and tumor samples

**Figure 1:**
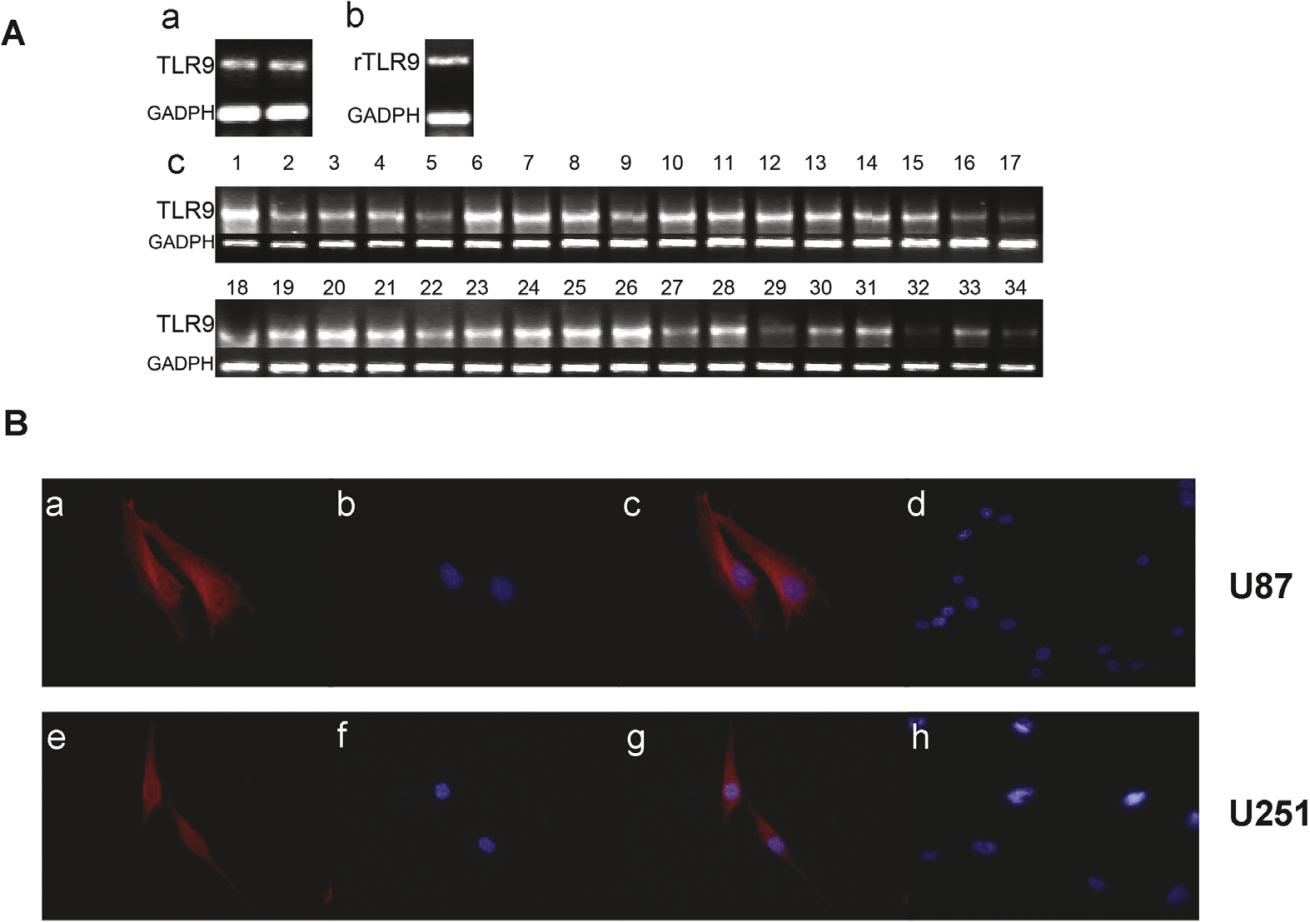
**Expression of TLR9 in glioma cell lines and glioma samples**. **A**. mRNA expression of TLR9 (a) Expression of TLR9 in human U87 glioma cell line(lane1) and human U251 glioma cell line(lane2); (b) Expression of TLR9 in rat glioma cell line C6. (c) Expression of TLR9 in 34 clinical glioma samples (lane1-15, Grade IV gliomas; lane16-28, grade III gliomas;lane 29-34, grade II gliomas). GAPDH was used as positive control. **B**. Protein expression of TLR9 in human glioma cell lines U87 and U251 with immunofluorescence staining. cytoplasmic localization of TLR9(red fluorescence, Cy3) in U87 and U251 was confirmed by confocal microscopy, nucleus was stained with Hoechst(Blue fluorescence). (a) TLR9 staining in U87 cells. (b) Hoechst staining of nucleus in U87 cells. (c) The combined figure of a and b. (d) negative control, staining of U87 cells with secondary antibody and Hoechst, but without primary anti-TLR9 antibody. (e) TLR9 staining in U251 cells. (f) Hoechst staining of nucleus in U251 cells. (g) The combined figure of e and f. (h) Negative control, staining of U251 cells with secondary antibody and Hoechst, but without primary anti-TLR9 antibody.

The expression of TLR9 mRNAs in glioma cell lines and tumor tissues were examined using RT-PCR method. As shown in Fig. 1A, TLR9 is expressed both in human and rat glioma cell lines, TLR9 expression in glioma tissues was also shown in Fig. 1A, all the 34 glioma samples tested in our experiment express TLR9. Housekeeping gene GAPDH was used as positive control.

Immunofluorescence was used to determine TLR9 protein expressed in human glioma cell lines. A cytoplasmic localization of TLR9 in U87 and U251 was confirmed by confocal microscopy (Fig. 1B). This finding is in agreement with previous studies on the distribution of TLR9 in RAW264.7 cells [19].

### Correlation between expression of TLR9 and malignancy of gliomas

**Figure 2:**
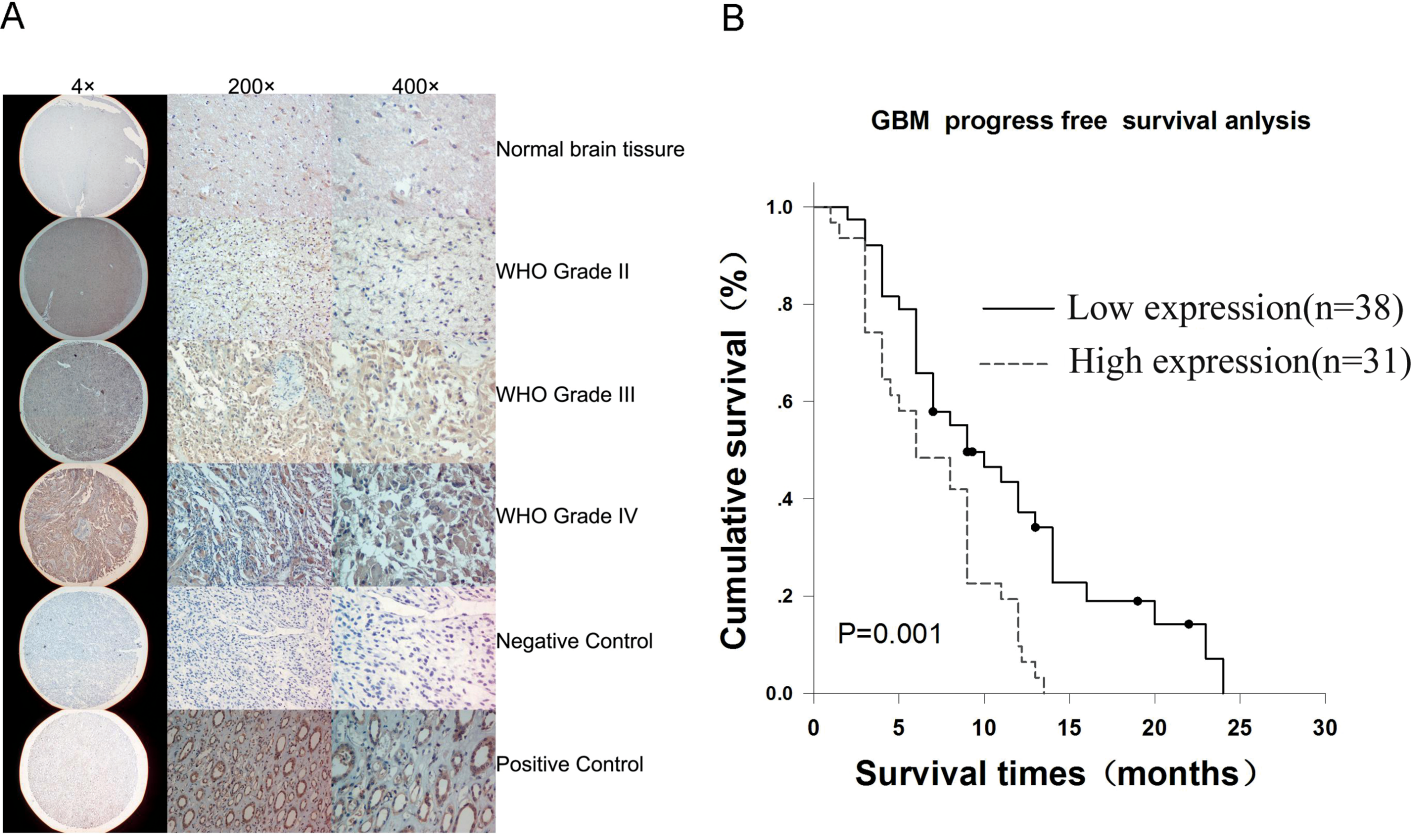
**TLR9 expression in glioma tissues correlated to glioma progression and the prognosis of GBM patients**. **A**. Representative immunohistochemistry staining photos of the glioma tissue arrays. Normal human kidney tissues provided by Department of Surgery were used as positive control. For negative controls, the primary antibody was replaced by normal mouse serum. Cell nuclei were counterstained with haematoxylin. **B**. Kaplan-Meier analysis showing the PFS(progress free survival) of glioblatoma (GBM) patients in low TLR9 expression group (bold line) and high TLR9 expression group (dotted line). The difference of PFS between the two groups is significant(p = 0.001).

To determine whether the expression level of TLR9 protein is associated with the histological characteristics of gliomas, glioma tissue microarray was constructed, sections were examined by immunohistochemical staining with an antibody against human TLR9. The anti-TLR9 antibody(Img-305A, clone 26C593.2, Imgenex, San Diego, USA) was widely used by other researchers. In our research, during immunohistochemistry staining, primary antibody control was replaced by normal mouse serum, which was widely used as negative control in immunoassay. Most importantly, we demonstrated that there is no TLR9 staining in normal brain tissues, which indicated no false positive staining exist in our study. It was demonstrated that kidney tubule but not the glomerus have the expression of TLR9[8], so we used kidney tubules as positive control for TLR9 staining in this study. As shown in Fig. 2A and Table 2, TLR9, mainly as cytoplasmic staining was found to be expressed in gliomas, no strong immunoreactivity (score <4) was detected in non-neoplastic brain tissues. TLR9 expression was detected in all high grade glioma cases and was highly expressed(score >4) in 43.78% (88/201) cases (Table 2). We next analyzed the correlation between the expression of TLR9 protein and the histological staging of gliomas. As summary in table 2, expression was significantly higher in high grade gliomas (grade III and grade IV) compared to low-grade gliomas (grade II) (P < 0.01), which supported that the TLR9 expression is associated with the progression of glioma.

**Table 2:** TLR9 expression is correlated with glioma progression

	Degree of TLR9 immunoreactivity (%)	
Characteristics	Low	High
Normal brain	13/13(100.00)	0/13(0. 00)
Low Grade^a^	80/91 (87.91)	11/91(12.09)
High Grade^b^	113/201(56.22)	88/201(43.78)
III^b1^	47/73(64.38)	26/73(35.62)
IV^b2^	66/128 (51.56)	62/128(48.44)

^a, b^P = 0.000; ^b1, b2^P = 0.078.

### Association between TLR9 expression and GBM patient prognosis

To determine the prognostic value of TLR9 protein for GBM patients, we assessed 69 GBM cases(Recurrent or dead before July 2009) using univariate Cox regression analysis(Table 3), we found that the expression levels of TLR9 protein were correlated with PFS of GBM patients (p = 0.013). As shown in Table 3, the multivariate analysis revealed that the expression levels of TLR9 protein level was a significant prognostic factor in GBM patients, independent of the conventional clinical variables (hazard ratio [HR], 3.205; 95% confidence interval [CI], 1.152-8.920; p = 0.026). In this study, age, gender, preoperative KPS, and extent of resection were not associated with PFS in GBM. We assessed the discriminative value of expression levels of TLR9 (low expression and high expression) for survival of patients with GBM using Kaplan-Meier analysis. We found that the expression levels of TLR9 could significantly discriminate the survival of the two GBM subgroups, with a mean PFS of 9 months (median PFS, 11.108 months; 95% CI, 8.843-13.372) for low TLR9 expression group versus mean 6 months (median PFS, 6.958 months; 95% CI, 5.634-8.283) for high TLR9 expression group (p = 0.001) (Fig. 2B and Table 3).

**Table 3:** Univariate and multivariate analysis of different prognostic parameters for PFS of GBM patients

Variables	Value	Univariate analysis	Multivariate analysis	
		***p***	**Hazard ratio (95% CI)**	***p***
Age*	44.96 ± 12.54	0.682	1.000 (0.982-1.018)	0.983
Gender				
Male	39(56.52%)	0.086	0.620 (0.238-1.617)	0.329
Female	30(43.48%)			
KPS*	78.00 ± 16.26	0.834	1.014 (0.996-1.033)	0.129
Extent of resection*	1.30 ± 0.77	0.149	1.456 (0.497-4.269)	0.174
TLR9 expression				
Low	38(55.07)	0.013	3.205 (1.152-8.920)	0.026
High	31(44.93)			

*Mean ± standard deviation

### CpG-ODN has no effect on glioma proliferation in vitro

**Figure 3:**
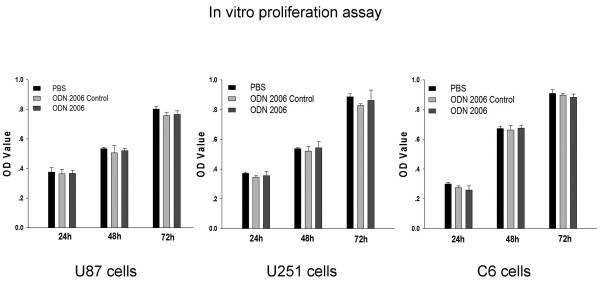
**Proliferation of glioma cell lines in response to CpG ODN(ODN 2006)**. Human U87 glioma cell line, human U251 glioma cell line, and rat C6 glioma cell line were incubated with CpG ODN(10 uM), CpG ODN Control(10 uM) or same volume of PBS. The analysis was performed in triplicate and cell growth was measured at 24, 48, 72 hours with an ELISA reader at 490 nm, data was represented as OD value. Bars refer to the standard error of the mean value. Comparison between groups was made by using student's t test, P ≤ 0.05 was defined as significant.

MTT assay were performed to investigate the effect of CpG ODN on the proliferation of glioma cells, glioma cell lines U87, U251 and C6 were used in this analysis, our results showed that CpG-ODN (ODN 2006) did not influence the proliferation rate of the three glioma cell line tested within 72 hours (Fig. 3), when compared with non CpG ODN (ODN2006 control) or PBS control. The ODN 2006 used in this research has been reported to be efficient activators of cells displaying mRNA expression of TLR9[20], and was widely used as a adjuvant for immunotherapy.

### CpG enhance glioma invasion in vitro

**Figure 4:**
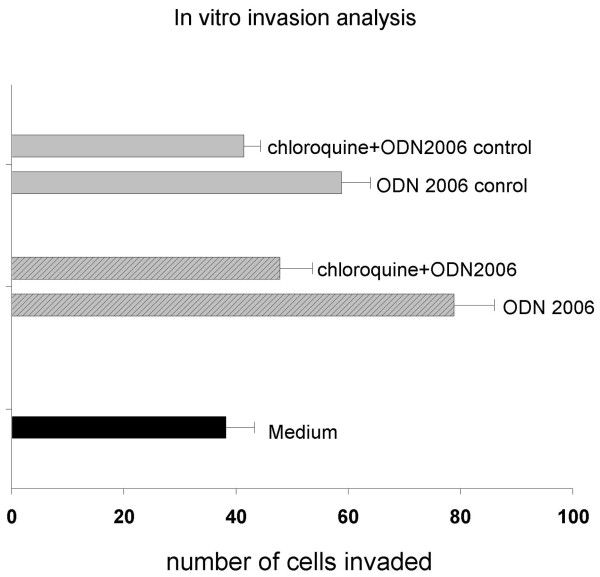
**Invasion of GBM cell line U87 in response to CpG ODN(ODN 2006)**. U87 cells were plated in 24 well cell culture chambers using transwell inserts precotated with matrigel. ODN 2006 (10 uM), ODN 2006 control (10 uM), same volume of the control medium, or TLR signaling inhibitor chloroquine (10 uM) were added and incubated for 24 hours, the number of invaded cells was stained and counted, all analysis were performed in triplicate. The invasion ability was represented as numbers of invaded cells. Bars refer to the standard error of the mean value. Comparison between groups was made by using student's t test, P ≤ 0.05 was defined as significant.

To study the effects of TLR9 stimulation on the invasion behavior of glioma cells, we performed invasion assays using the well characterized TLR9 agonist, ODN2006. As shown in Fig. 4, the invasion of glioma U87 cells was significantly elevated by CpG ODN(ODN2006), compared with that of glioma cells treated with medium. Similar to other researchers' results [11], the non-CpG ODN(ODN2006 control) can also enhance the invasion of U87 cells. The increased cell numbers seen in the invasion assays were not due to effect of proliferation, because the ODN had no effects on cell viability(Fig. 3). In order to ensure that TLR9 is responsible for the invasion effects induced by CpG ODN, glioma cells were treated with CpG ODN in the presence of chloroquine, which is an inhibitor of endosomal acidification resulting in inhibition of TLR9 signaling[21]. As shown in Fig. 4, chloroquine significantly inhibited CpG ODN induced invasion in glioma cells, which suggest that TLR9 signaling is responsible for the enhanced invasion of U87 cells induced by CpG ODN.

## Discussion

Glioma is the most frequent tumor originating from glial cells in the nervous system, various glioma subtypes has been divided by grade or by the cell type. Each subtype constitutes different genetic mutations[22] and likely differ in prognosis and response to therapy. We proposed that there may exist some specific biological markers related to the clinical behaviors of gliomas, these markers may also involved in signal pathways that driving the initiation and development of gliomas.

Recently, researchers showed that virus may be responsible for the initiation and development of glioma[23-25]. Mitchell et al[25] found a high percentage (more than 90%) of GBM tumors, not surrounding normal brain, are associated with HCMV nucleic acids and proteins. Thus a virus or inflammation related biomarker may be predictive of glioma progression and development. It was demonstrated that HCMV infection strongly induced TLR9 expression in fibroblast[26]. TLR9 belongs to Toll like receptors, which are a family of pattern recognition receptors that sense highly conserved structures from microorganisms [27]. Among the TLR family, TLR9 recognizes the ODN with CpG motif. After binding with the ligand, TLR9 signal pathway leading to subsequent downstream activation of the NFκB, and MAPK signaling pathways[28], which may responsible for the proinflammatory or progrowth microenvironment of tumor. Recent studies suggested that many different tumor cell types, such as colon[29], prostate[10], breast[7], lung[9] et al express TLR9 and that TLR9 signaling promotes tumor growth, survival and immune evasion. Thus we proposed that The TLR9 pathway may also exist and activated in glioma tissues.

There are controversy results for the expression of TLR9 in glioma cells, Grauer et al[13] showed that TLR9 are essentially absent at GL261 cells, While Andaloussi et al [14] found a strong expression of TLR9 in GL261 cells with RT-PCR and FCM analysis. This difference was most likely not caused by the contamination of genomic DNA at RT-PCR analysis, since Andaloussi et al [14]further confirmed their RT-PCR result with FCM. We preferred Grauer's data because their result is consistent with the function of TLR9 signal pathway. As a receptor for CpG ODN, TLR9 play a key role in tissue repair and cancer progression, it was demonstrated that CpGs stimulated the invasion of TLR9+ cancer cells, but not TLR9- cancer cells[11]. Grauer et al [13]showed that GL261 is TLR-, which indicated that CpG ODN may not affect the invasion of GL261 cells, this was supported by widely accepted results which indicated that CpG ODN induced apoptosis of GL261 in vitro and cured GL261 glioma animal model in vivo.

Meng et al [17]and Andaloussi et al [14]found that U87 and U251 express high level of TLR9 mRNA, however no data about the expression of TLR9 protein in these two cell lines was reported. To avoid the controversy data like GL261, we investigated the expression of TLR9 in U87 and U251 cells at both the mRNA level(RT-PCR) and protein level(immunofluoresence), our result for the first time showed that TLR9 staining located intracellularly and could be seen throughout the cytoplasm in U87 and U251 glioma cells.

The relationship between the expression level of TLR9 and tumor grade of glioma is unknown, to investigate this, large amount of clinical samples are required. Meng et al [17]checked the expression of TLR9 in 37 frozen glioma samples, and they found no relationship between the TLR9 expression and survival, however, there are several factors may affect their results in their study: 1. some of the their samples were frozen for a very long time since 1990. 2. their samples were frozen under -180°C, the frozen and thawing process may damage the cells and thus affect the result of immunohistochemistry analysis. 3. for the QPCR experiment, they set a QPCR threshold(0.025) to divide the patients into low TLR9 group and high TLR9 group, however, this threshold is not a widely accepted criteria and may affect their result. 4. Only quantitative RT-PCR data was used and there is no quantitative protein level data, the non-neoplastic brain tissues and necrotic tissue, which have low expression of TLR9, may be included in the tissues for the extraction of mRNA from the specimen, and thus may interfere with the result. Because of these reasons, their results require confirmation with larger specimen numbers.

We screened the expression of TLR9 in a large collection of human glioma samples in tissue array with immunochemistry. With the mutivariant analysis(Table 3), we found that TLR9 expression is an independent prognostic factor to predict patient PFS in GBM (P <0.05), which indicated that the expression levels of TLR9 correlate significantly with the clinical outcome of patients with GBM. Other biological factors such as age, gender, KPS before surgery, the extent of resection were not found associated with prognosis. Our research include same group of patients that was used in one previous published study[30], which showed that the resection extent and KPS were not related to the survival, and these data were further supported by other researcher's results. Based on the published literature, it remains unclear whether the extent of surgical resection correlates with survival [31-33]. Even for KPS, there are many studies found that it is not associated with survival of GBM patients[34-36]. In addition to this, our study for the first time demonstrated that TLR9 expression increased significantly from low grade (grade II) glioma to high grade (grade III and grade IV) glioma(Table 2), this result is similar to results obtained by other researchers with clinical breast specimen[37] and cervical neoplasia[38]. Present data indicated that TLR9 expression increases according to the histopathological grade of glioma, and the TLR9 expression level is related to the PFS of GBM patients.

The potential relationship between TLR9 pathway and cell proliferation was explored, we demonstrated that TLR9 activator CpG ODN did not affect the proliferation of glioma cells in vitro. Merrell et al [11]showed that treatment of TLR9 expressing breast and brain cancer cells(U373 and D54MG) with CpG ODN stimulates their invasion via MMP 13 activation. In our research, a different glioma cell line(U87 cells) were used to ensure whether or not TLR9 is responsible for the invasive of glioma cells induced by CpG ODN. We believe that, just like the TLR9 signal pathways in other cancer cells, the activation of TLR9 may lead to enhanced invasiveness of U87 glioma cells, this was supported by our research, we found that the TLR9 agonist CpG ODN(ODN 2006) can significantly increased the invasiveness of U87 glioma cells(Fig. 4). Most importantly, we found that the CpG induced invasion could be blocked with chloroquine, which is an inhibitor of TLR9 signaling pathway. All the results suggested that the expression of TLR9 in glioma cells is functional and related to the invasiveness of glioma cells. In this research, we found that non-CpG ODN(ODN2006 control) can also increase the invasion of glioma cells through TLR9 signaling pathway, which indicated that non-CpG ODNs (ODNs from *in vivo *cells such as dying tumor cells) may also be able to increase the invasion of glioma through TLR9 pathway. In addition to this, for the first time, we showed that chloroquine was able to inhibit the TLR9 induced invasiveness of glioma cells(Fig. 4), and thus may be used as an adjuvant for the GBM therapy, this was confirmed by Briceno et al[39,40], who showed that chloroquine may improve mid-term survival when given in addition to conventional therapy for GBM. However, the exact mechanism for the TLR9 regulation of glioma invasion need further investigation and is out of the focus of this research.

Our finding has several important implications: First, they may give novel clues for the investigation of the mechanism of glioma initiation and development. Secondly, our findings warrant caution in the directly injection of TLR9 agonist CpG ODN into glioma tissues for the glioma immunotherapy., this conclusion may be contradictory to some researcher's work[13,41,42]. Although there is contradictory results for the expression of TLR9 in the GL261 cells between Grauer's result[13] and Meng's result[17], both of them confirmed that the prolonged survival seen in their study was not due to the expression of TLR9 expression in glioma cells, but rather due to increased apoptosis of GL261 cells in vivo and enhanced modulation of the local CNS immune response. TLR9 expression in non-tumoral cells or microglia activation may play an important role in the CpG induced anti-cancer immunotherapy, however, the inflammatory response observed following CpG treatment do not always prevent growth of solid tumor masses, in one CpG treated glioma animal model[43], intratumoral application of CpG proved ineffective. In addition to this, Deng et al[44] showed that intracerebral injection of ODN containing CpG motif induce meningitis in mice and rat. In a phase II trial evaluating the efficacy of CpG in 34 recurrent GBM patients[45], patients usually experienced transient neurological worsening or fatigure after intratumoral application of CpG. In 3 patients, seizure occurred just after administration of CpG. Our research indicated that locally administered CpG ODN may increase the invasion of glioma cells. Based on these data, carefully safety evaluation in animal model and in large amount of glioma patients should be performed before CpG can be routinely intratumoral administered in clinic. For the glioma immunotherapy strategy with intratumoral injection of CpG ODN, the most successful animal model is GL261 mouse model[13,14]. However, we think GL261 was not a perfect glioma model, since CpG ODN can inhibit GL261 cell proliferation in a cell type specific manner[13], but for human cell lines such as U87 and U251, or rat glioma cell line such as C6, we did not find such effect in this study(Fig. 3). So, it is difficult to translate the data from GL261 model into other cell lines or other models. We did not deny the effectiveness of CpG ODN on tumor immunotherapy, our previous experiment demonstrated that the CpG ODN can activate immune system systemically and cure the brain tumor when it was delivered subcutaneously [16]. We think CpG ODN may activate TLR9 and promote tumor progression if it was delivered locally into the tumor within the brain. CpG ODN may be a magic antitumor weapon, but how to use it and where to use it is important question, which may lead to very different result.

We must emphasis that we did not think the TLR9 only express in glioma cells, the infiltrated lymphocyte, the microglial cells may also express high level of TLR9, Meng et al[17] indicated that TLR9 was mainly expressed in infiltrated lymphocyte or microglial cells in GBM tissues. However, in our study, we found the glioma cells do express TLR9(Fig. 1). We consider the glioma tissue as an unseperatabe whole, which was consist of glioma cells, infiltrated lymphocytes and microglial cells et al. The expression of TLR9 in different kind of cells within the glioma tissue, but not only in glioma cells, will affect the prognosis of patients with glioma., we think the TLR9 expression may reflect inflammatory microenviroment within glioma tissues.

There may be several reasons for the high expression of TLR9 in GBM tissues: 1. by stander phenomenon, the promoter of TLR9 was activated because of other activated pathways in GBM. 2. The upregulation of TLR9 could be beneficial to the tumor, promoting survival and the invasiveness of glioma cells, the selective pressure of the local microenvironment might result in the ultimate higher expression of TLR9. 3. The presence of HCMV in gliomas[24] may responsible for the high TLR9 expression, however, the relationship between the CMV and TLR9 expression need further investigation. At present time, the relationship between inflammation and tumorigenesis is widely accepted. However, the cellular and molecular mechanism involved in this process is incompletely understood. Our study indicated that the TLR9 signaling pathway constitute an important cellular pathway mediating this interaction in glioma, however, other independent retrospective and prospective studies will be necessary before direct clinical application of the current findings. The exact pathophysiological role of TLR9 in glioma is not known but it may represent a useful prognostic biomarker in glioma patients. The expression of TLR9 in glioma tissues is a major factor, through them, glioma cells can recognize either microbial pathogens or cellular debris and promote the expression and secretion of chemokines and cytokines. We demonstrated the relationship between TLR9 expression and glioma progression. Better understanding of the function and regulation of the TLR signaling pathways in glioma may shed new lights on understanding the mechanisms of glioma formation and progression, as well as provide new targets for more effective regimens to treat gliomas.

## Conclusions

TLR9 recognizes the ODN with CpG motif. After binding with the ligand, TLR9 signal pathway may induce the proinflammatory or progrowth microenvironment of tumor. Our data indicated that TLR9 is expressed in glioma cell lines and glioma tissues, and the expression of TLR9 in glioma cells is functional. CpG ODN can significantly enhance the invasion of GBM cells, we confirmed that TLR9 signaling was responsible for the enhanced invasion of glioma cells induced by CpG ODN. Screening a large collection of human glioma samples in tissue array with immunohistochemistry, we found that TLR9 expression correlated to the malignancy of gliomas, and TLR9 expression is an independent prognostic factor to predict patient PFS in GBM. Our data indicated that TLR9 may relate to glioma progression and the prognosis of GBM patients. In addition, our findings warrant caution in the directly injection of TLR9 agonist CpG ODN into glioma tissues for the glioma immunotherapy.

## Competing interests

The authors declare that they have no competing interests.

## Authors' contributions

WC: carried out the tissue array analysis, interpreted the data and drafted the manuscript; CS: Carried out the cell culture and immunoflurecence staing; YY: carried out the RT-PCR analysis; QY: supported in the tissue collection and statistical data analysis; JT: supported in the tissue collection; XK: participated in design and coordination of the study; WA: conceived and designed the study, participated in study coordination, data analysis, data interpretation and drafting of the manuscript. All authors read and approved the final manuscript

## Pre-publication history

The pre-publication history for this paper can be accessed here:

http://www.biomedcentral.com/1471-2407/10/415/prepub
